# Word and Works

**Published:** 1889-07

**Authors:** 


WORD AND WORKS.—We are pleased to welcome on our exchange list the paper known as *Word and Works*, published by the Rev. Irl R. Hicks of St. Louis, Mo. The forecasts of weather by Mr. Hicks, which are regularly published in his paper have proven accurate beyond anything heretofore known in the past history of the world. On this account, and the additional interesting moral and scientific information contained in it, his paper is becoming very popular with the reading public.

